# Phylogenetic analysis of H5N1 influenza viruses isolated from dairy cattle in Texas in December 2024

**DOI:** 10.1128/jvi.00580-25

**Published:** 2025-07-08

**Authors:** Sangam Kandel, Lavanya Babujee, Lizheng Guan, Randall Dahn, David Pattinson, Peter J. Halfmann, Amie J. Eisfeld, Gabriele Neumann, Alexis C. Thompson, Ellen Ruth Alexander Morris, Amy K. Swinford, Keith Poulsen, Kiril M. Dimitrov, Yoshihiro Kawaoka

**Affiliations:** 1 Department of Pathobiological Sciences, Influenza Research Institute, University of Wisconsin-Madison 5228, Madison, Wisconsin, USA; 2 Texas A&M Veterinary Medical Diagnostic Laboratory 117328 , Canyon, Texas, USA; 3 Texas A&M Veterinary Medical Diagnostic Laboratory 117328 , College Station, Texas, USA; 4 Wisconsin Veterinary Diagnostic Laboratory, University of Wisconsin-Madison 5228, Madison, Wisconsin, USA; 5 Department of Virology, Institute of Medical Science, University of Tokyo 26430 , Tokyo, Japan; 6 The University of Tokyo Pandemic Preparedness, Infection and Advanced research center (UTOPIA) University of Tokyo 13143, Tokyo, Japan; 7 The Research Center for Global Viral Diseases, National Center for Global Health and Medicine Research Institute, Tokyo, Japan; St. Jude Children's Research Hospital, Memphis, Tennessee, USA

**Keywords:** highly pathogenic, H5, dairy cattle, influenza virus

## LETTER

Highly pathogenic avian influenza (HPAI) viruses of genotype B3.13 were first reported in dairy cattle in March 2024 and, by 4 March 2025, had infected at least 989 cattle herds in 17 states in the US (https://www.aphis.usda.gov/livestock-poultry-disease/avian/avian-influenza/hpai-detections/hpai-confirmed-cases-livestock; accessed on March 24, 2025). Importantly, spillover infections of poultry and mammalian species, including cats and humans, have occurred (https://www.cdc.gov/bird-flu/situation-summary/index.html; accessed on 4 March 2025). Texas reported several infected herds in the spring (1) and early summer of 2024 but saw fewer outbreaks during the second half of 2024, with reported outbreaks in July and December (2). Since September 2024, almost all reported outbreaks of genotype B3.13 viruses in dairy cattle have occurred in California. Here, we characterized self-submitted samples from a farm in Texas that experienced an outbreak in December 2024. This farm had purchased dairy cattle from several states in the US. We, therefore, asked whether the December 2024 outbreak in Texas was caused by B3.13 viruses that circulated undetected in dairy cattle or other species in Texas or resulted from the introduction of B3.13 viruses from California (having the highest number of affected farms at that time) or other states.

**Fig 1:**
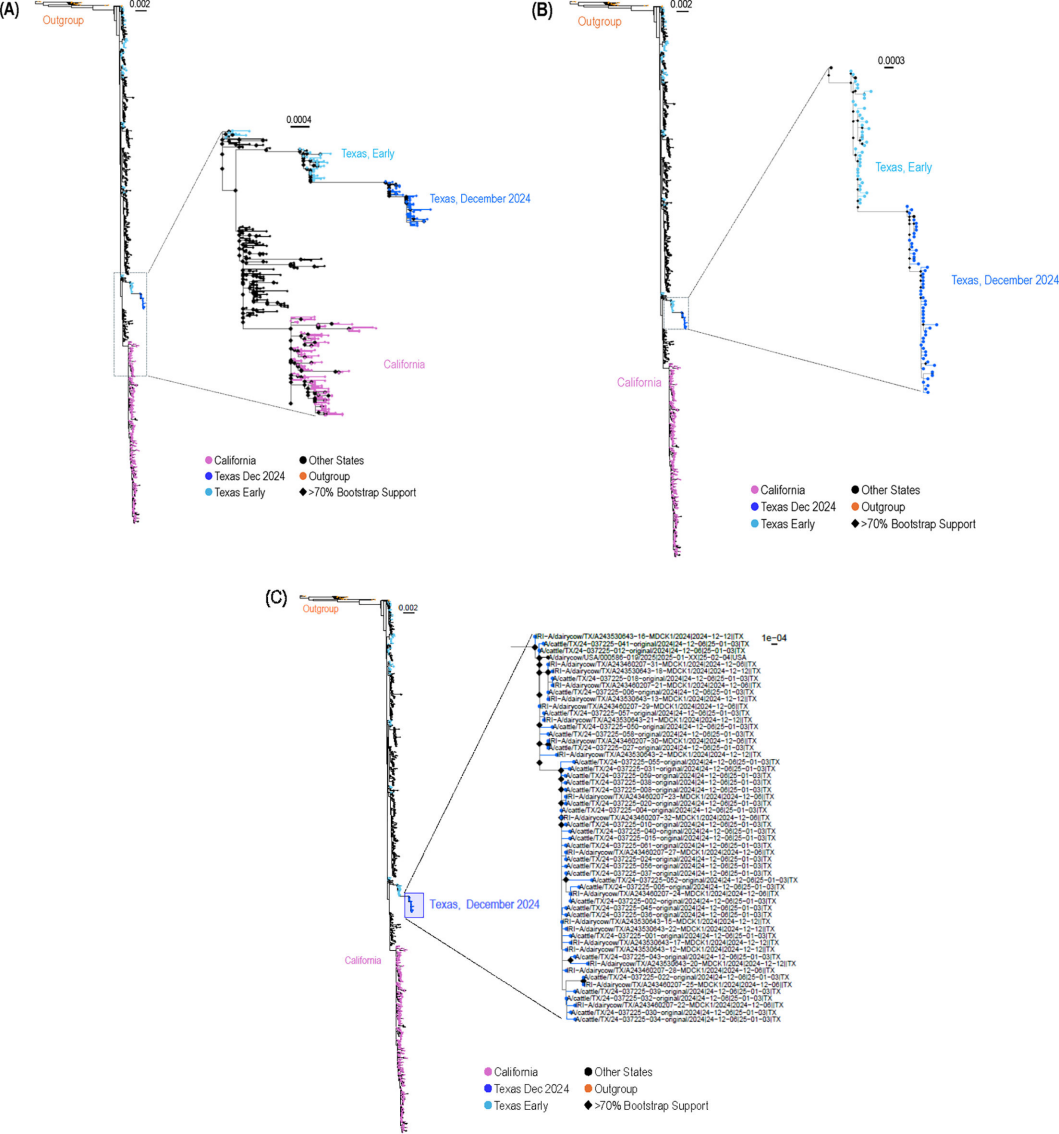
Maximum likelihood (ML) phylogeny of H5N1 viruses collected from dairy cattle in Texas, USA in December 2024. Nucleotide sequences for all eight segments of all North American H5N1 clade 2.3.4.4b viruses were downloaded from NCBI Virus (https://www.ncbi.nlm.nih.gov/labs/virus/vssi/#/) and GISAID (https://gisaid.org/; Table S2) on 5 February 2025. Viruses with unknown collection locations or sampling dates were removed, as were duplicate sequences. Sequences were aligned by using MAFFT (8) (v7.490), and alignments were hand curated to remove gaps introduced by the alignment process. Sequences with >9 undetermined bases were removed from further analysis. For inferring phylogeny, the sequences were concatenated to generate genomes, and unique sequences were retained. To this set, genomes of viruses generated in this study (GenBank Accessions xxx; indicated in the tree by the prefix “IRI”) and genomes of an outgroup set of 20 avian influenza viruses closely related to the B3.13 viruses that infected dairy cattle were added. An ML tree was generated by using the GTR + F + I + G4 model in RAxML-NG (v. 1.1.0) (4). Node support was evaluated by up to 1,000 bootstrap replicates using the extended majority rules (MRE)-based bootstrapping (9). For the December 2024 viruses from Texas, high bootstrap values (>70%) are indicated by black diamonds. In panels **A**, **B**, and **C**, the viruses characterized here are increasingly zoomed in.

We received 38 milk samples from the Texas A&M Veterinary Medical Diagnostic Laboratory (collected on 6 December 2024 and 8 December 2024) from which 21 viruses were isolated (Table S1). All viruses were amplified in MDCK cells and embryonated chicken eggs. The virus stocks were then sequenced on the Oxford Nanopore MinION flow cell (FLO-MIN114) using Native Barcoding Kit 96 V14 (SQK-NBD114.96). Reads were preprocessed and assembled *de novo* using the iterative meta-assembler (IRMA, v.1.0.2) (3) within a Snakemake workflow v1.0.1 (https://github.com/IRI-UW-Bioinformatics/flu-ngs) to establish consensus sequences; the minimal sequence depth was ~34,500 reads. Maximum likelihood phylogenetic analysis of concatenated, MDCK cell-grown virus genomes was conducted by using RAxML-NG (v 1.1.0) (4) with the GTR + F + I + G4 model (Fig. 1). The isolated viruses are closely related to B3.13 viruses isolated in Texas in the summer of 2024, supported by bootstrap values of >0.7 (Fig. 1). Phylogenetic analysis of individual viral gene segments demonstrated a close relationship between B3.13 viruses isolated in July and those isolated in December (Fig. S1). Compared to April 2024 viruses from Texas, the proteins of the December 2024 viruses accumulated the following amino acid substitutions: PB2-E249D, -I266V, and -V344M; PB1-I164M; PA-A20V and PA-K603R; HA-V147M, -L338P, NA-S21G, -P37S, -V116I, -S369R, -R399W, -T435A, and -R469K; and NS1-E55A. As examples, the phylogenetic trees of HA, NA, and NP are shown in Fig. S2. Amino acid position 147 of HA (position 131 of mature HA) is located at the rim of the receptor-binding site so that the HA-V147M substitution might affect receptor-binding and/or antigenicity. Amino acid position 338 of HA (position 322 of mature HA) is located upstream of the multibasic cleavage site of HA. The consequences of the HA-L338P substitution are not known. Some of the NA substitutions are located close together on the 3D structure (Fig. S3). The amino acids at positions 369 and 399 are sialic acid contact residues in the second sialic acid-binding site of NA, which comprises amino acids 363–370, 395–400, and 430–433 (based on the NA amino acid sequence of the viruses characterized here) (5). Interestingly, mutations in the second sialic acid-binding site of NA (although at a different amino acid position) were also detected in HPAI H5N1 viruses isolated from infected mink in Spain in 2022 (6). These mutations may affect the sialic acid binding of NA (7). Collectively, our analysis indicates that genotype B3.13 viruses continuously circulated in Texas, even though no outbreaks were reported between July and December 2024. It is unclear if the viruses were maintained in dairy cattle (apparently healthy or undiagnosed), or if they spilled over into other species and were reintroduced into dairy cows.

### Biosafety statement

The isolation of the HPAI B3.13 viruses was carried out in biosafety level 3 (BSL-3) containment laboratories at the Influenza Research Institute at the University of Wisconsin–Madison, which is approved by the Federal Select Agent Program for studies with these viruses. Funding for this study came in part from the NIAID Centers of Excellence for Influenza Research and Response (Contract Number 75N93021C00014). The NIAID grant for the studies conducted was reviewed by the University of Wisconsin–Madison Dual Use Research of Concern (DURC) Subcommittee in accordance with the United States Government September 2014 DURC Policy and determined to not meet the criteria of DURC. The University of Wisconsin–Madison Institutional Contact for Dual Use Research reviewed this manuscript and confirmed that the studies described here do not meet the criteria of DURC.

## Data Availability

The sequences of the viruses characterized here are available at GenBank under accession numbers PV864976-PV865031, PV865033-PV865040, PV865042-PV865049, PV865051-PV865058, PV865060-PV865091, and PV865093-PV865148. Our workflow for sequence analysis is described at https://github.com/IRI-UW-Bioinformatics/flu-ngs.

## SUPPLEMENTAL MATERIAL

Supplemental figures jvi.00580-25-s0001.pdf Figures S1 to S3.

Supplemental text jvi.00580-25-s0002.docx Supplemental methods.

Table S1 jvi.00580-25-s0003.xlsx Bovine H5N1 viruses isolated from milk samples collected in Texas, December 2024.

Table S2 jvi.00580-25-s0004.xlsx Originating laboratories and the submitting laboratories where genetic sequence data originated.

ASM does not own the copyrights to Supplemental Material that may be linked to, or accessed through, an article. The authors have granted ASM a non-exclusive, world-wide license to publish the Supplemental Material files. Please contact the corresponding author directly for reuse.
