# Supplementary material for: Phylogenetic analysis of H5N1 influenza viruses isolated from dairy cattle in Texas in December 2024
Source: J Virol. 2025 Jul 8;99(8):e00580-25. doi: 10.1128/jvi.00580-25 (PMC12363181; doi:10.1128/jvi.00580-25)
Supplement: Supplemental figures — Figures S1 to S3. [file jvi.00580-25-s0001.pdf]

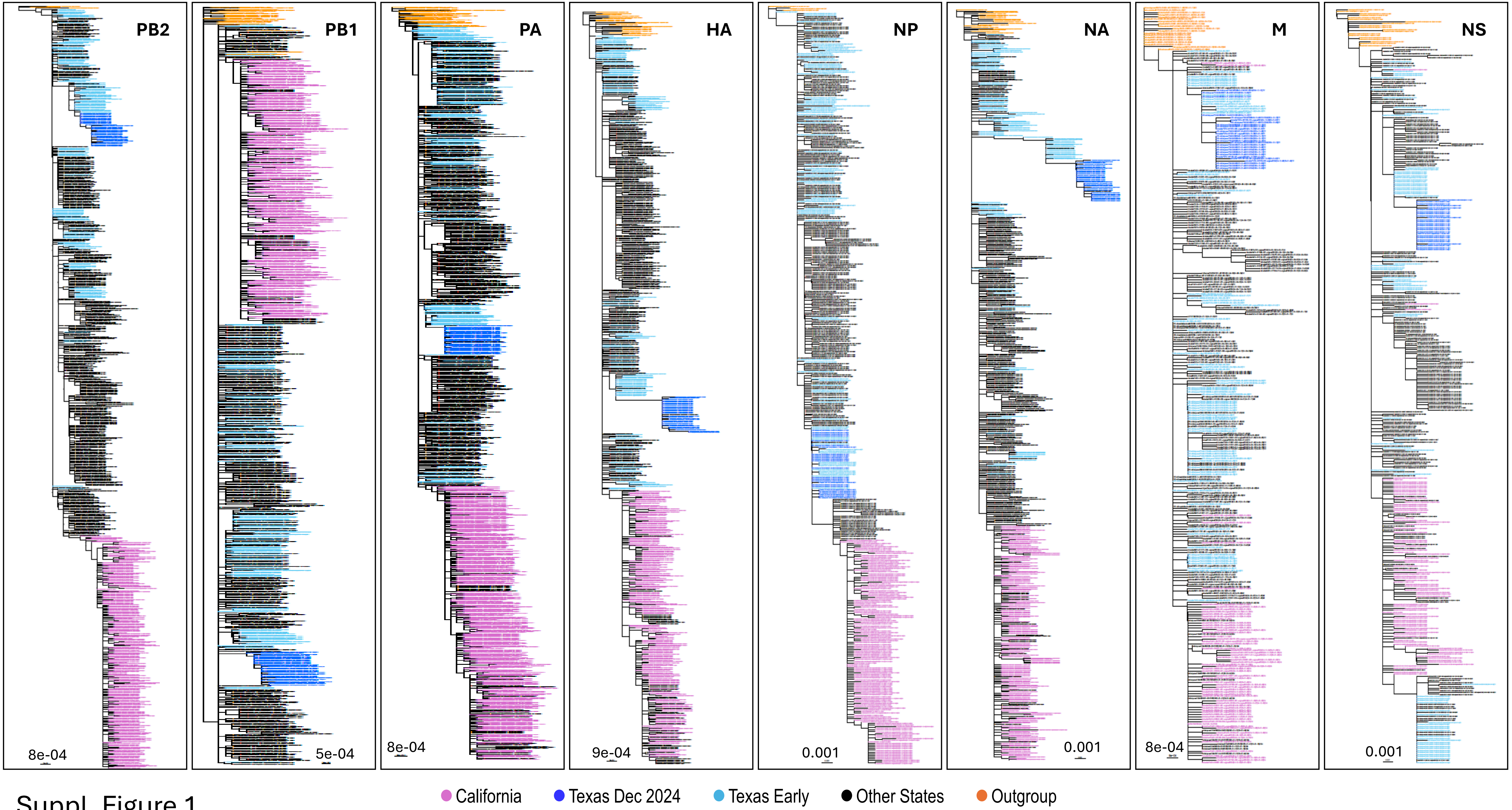

Suppl. Figure 1

Supplementary Figure 1. Maximum likelihood phylogeny of individual H5N1 virus gene segments of dairy cattle virus from Texas, USA in December 2024. Maximum likelihood phylogeny for individual gene segments was carried out as described in Figure 1. For the PB2 and NP segments, a subset of the outgroup set was used. For most nodes, bootstrap values were ≤70%.

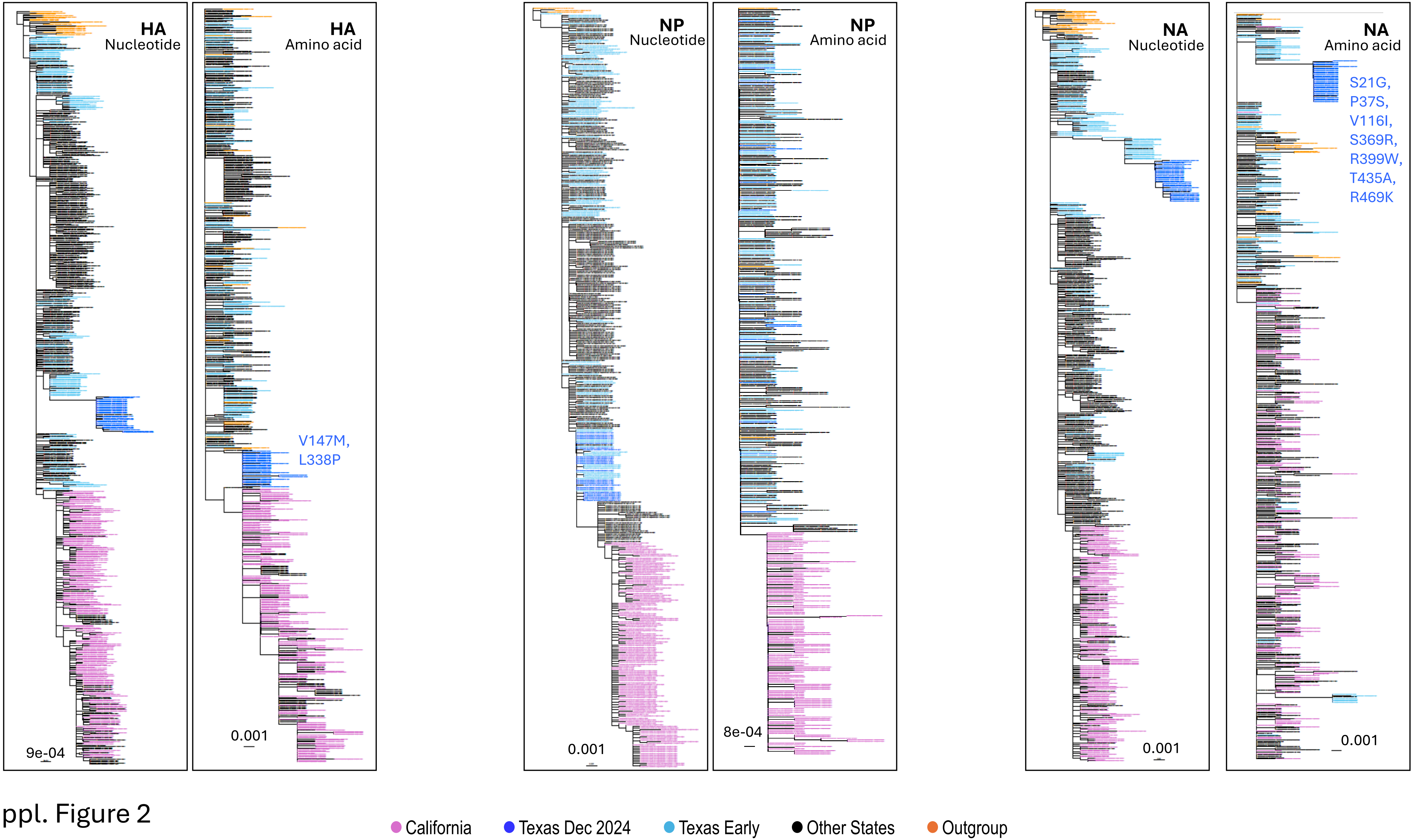

Suppl. Figure 2

Supplementary Figure 2. Maximum likelihood phylogeny of H5N1 HA, NP, and NA virus proteins of dairy cattle virus from Texas, USA in December 2024. Maximum likelihood phylogeny for individual proteins was carried out on amino acid sequences as described in Figure 1, with the FLU+F+I model. For NP, a subset of the outgroup set was used. For HA and NA, the amino acid differences between April and December 2024 isolates from Texas are shown. For most nodes, bootstrap values were ≤70%.

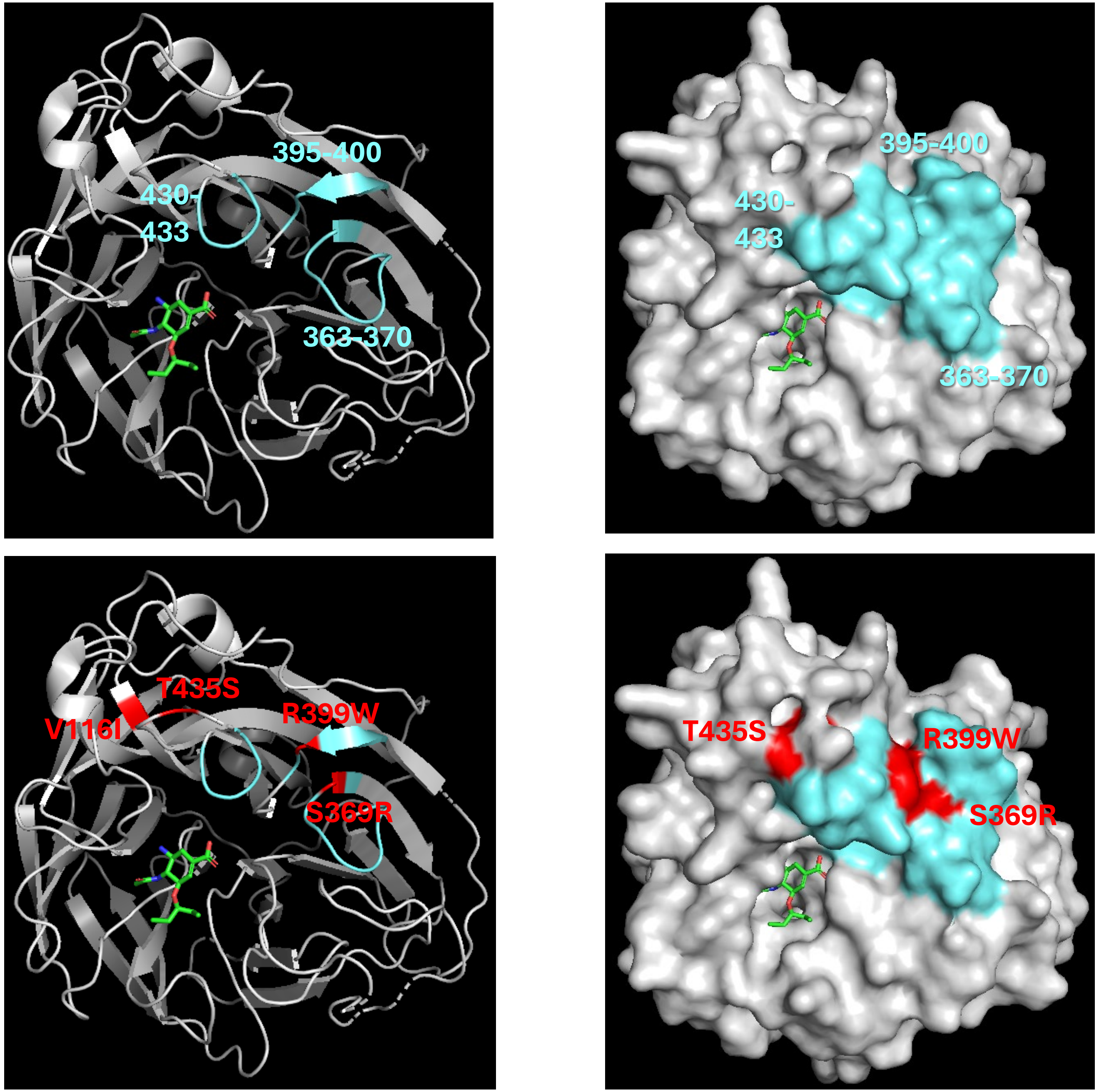

Suppl. Figure 3

Supplementary Figure 3. Three-dimensional structure of N1 NA with oseltamivir carboxylate (protein database accession #3CL0). Shown in red are the amino acid locations and substitutions characteristic of December 2024 viruses from Texas, compared to those isolated in April (see Suppl. Figure 2). Shown in teal is the location of the second sialic acid-binding site in NA [2]. Note that amino acid positions 21, 37, and 469 are not resolved in the structure.
