# Supplementary material for: Phylogenetic analysis of H5N1 influenza viruses isolated from dairy cattle in Texas in December 2024
Source: J Virol. 2025 Jul 8;99(8):e00580-25. doi: 10.1128/jvi.00580-25 (PMC12363181; doi:10.1128/jvi.00580-25)
Supplement: Supplemental text — Supplemental methods. [file jvi.00580-25-s0002.docx]

**Supplemental Methods**

**Next-generation sequencing.**

Viral RNA was extracted from infected MDCK cells and the allantoic fluid of embryonated chicken eggs using the QIAamp Viral RNA Mini kit (Qiagen), following manufacturer’s instructions. Whole genome RT-PCR amplification of the viral RNA was performed using SuperScript™ IV One-Step RT-PCR System (Invitrogen, USA) with primers described previously^1^ . The primers used were: HFadapter: 5′-TCGTCGGCAGCGTCAGATGTGTATAAGAGACAGAGCRAAAGCAGG;
HF: 5′- TGTATAAGAGACAGAGCRAAAGCAGG;
HRadapter: 5′-GTCTCGTGGGCTCGGAGATGTGTATAAGAGACAGAGTAGAAACAAGG;
HR: 5′- TGTATAAGAGACAGAGTAGAAACAAGG;

To improve the amplification of polymerase segments in some of the samples, we used segment- specific primers as listed below: H5N1-PB2-F: 5′-AGCGAAAGCAGGTCAAATATATTCA;

H5N1-PB2-R: 5′-AGTAGAAACAAGGTCGTTTTTAAACAATTC;

H5N1-PB1-F: 5′-AGCGAAAGCAGGCAAACCATTT;

H5N1-PB1-R:5′-AGTAGAAACAAGGCATTTTTTCATGAAGGA;

H5N1-PA-F: 5′-AGCAAAAGCAGGTACTGATTCAAAATG;

H5N1-PA-R: 5′-AGTAGAAACAAGGTACTTTTTTGGACAGTA .

Samples with gaps in the polymerase segments were reamplified by synthesizing tiled amplicons with the primers listed below:

| **Name** | **Sequence** | **Pool** |
| --- | --- | --- |
| H5N1-PB2-1-LEFT | GTCACAGTCTCGCACTCGC | 1 |
| H5N1-PB2-1-RIGHT | GTATGCAACCATCAGAGGGGC | 1 |
| H5N1-PB2-2-LEFT | CTGACCTCAGCGCCAAAGAG | 2 |
| H5N1-PB2-2-RIGHT | TTCAATGTTTGAAGGTTGCCCG | 2 |
| H5N1-PB2-3-LEFT | CCGTGGACATATGCAAGGCA | 1 |
| H5N1-PB2-3-RIGHT | CCCGTTGGTCTCGAACTCTT | 1 |
| H5N1-PB2-4-LEFT | GGGAATGATCGGGATATTGCCTG | 2 |
| H5N1-PB2-4-RIGHT | GCCTCTTGGTGGCCTTGTTG | 2 |
| H5N1-PB2-5-LEFT | TTCAATGGTCACAAGATCCCACAA | 1 |
| H5N1-PB2-5-RIGHT | GCCATCCGAATTCTTTTGGTCG | 1 |
| H5N1-PB1-1-LEFT | AGTTCCAGCGCAAAATGCCA | 1 |
| H5N1-PB1-1-RIGHT | GTCTCTCACTCTTCTTTTCCTTTGGA | 1 |
| H5N1-PB1-2-LEFT | AATCAGCCGGCTGCAACTG | 2 |
| H5N1-PB1-2-RIGHT | GGATTCTGGTTCTCATTCCATTTAGTG | 2 |
| H5N1-PB1-3-LEFT | TGCAAATCAGAGGGTTTGTGTAC | 1 |
| H5N1-PB1-3-RIGHT | TGTTTTGGTGTACTTCTTTTGCCC | 1 |
| H5N1-PB1-4-LEFT | CCTCTTCTAATAGATGGTACGGCC | 2 |
| H5N1-PB1-4-RIGHT | CAGCTCGAATGACCTCCTTGT | 2 |
| H5N1-PB1-5-LEFT | GGACCAGCAACAGCCCAAA | 1 |
| H5N1-PB1-5-RIGHT | GCCTCCACCATGCTGGAAAT | 1 |
| H5N1-PA-1-LEFT | GGAAGACTTTGTGCGACAATGC | 1 |
| H5N1-PA-1-RIGHT | GACGAAAGGAATCCCATAGACCTC | 1 |
| H5N1-PA-2-LEFT | CACATATTCTCATTCACTGGAGAGGA | 2 |
| H5N1-PA-2-RIGHT | TGCGGCTTTACGATGTTGGG | 2 |
| H5N1-PA-3-LEFT | AGCGGTCGAAGTTCTTGCTG | 1 |
| H5N1-PA-3-RIGHT | TGTATTCAGTGGCCCTGCAA | 1 |
| H5N1-PA-4-LEFT | CAACAAGGCATGCGAACTGAC | 2 |
| H5N1-PA-4-RIGHT | CGGCCTCAATCATGCTCTCAA | 2 |
| H5N1-PA-5-LEFT | AACTGACCCGAGGCTAGAGC | 1 |
| H5N1-PA-5-RIGHT | GTGCATGTGTGAGGAAGGAGTT | 1 |

**Supplemental references.**

1. Lee HK, Lee CK, Tang JW, Loh TP, Koay ES. Contamination controlled high-throughput whole genome sequencing for influenza A viruses using the MiSeq sequencer*. Sci Rep*. 2016;6:33318.
2. Du W, de Vries E, van Kuppeveld FJM, Matrosovich M, de Haan CAM. Second sialic acid-binding site of influenza A virus neuraminidase: binding receptors for efficient release. *FEBS J* 2021; **288**(19): 5598-612.
